# Reference ranges for organ weights of infants at autopsy: results of >1,000 consecutive cases from a single centre

**DOI:** 10.1186/1472-6890-14-18

**Published:** 2014-04-28

**Authors:** Jeremy W Pryce, Andrew R Bamber, Michael T Ashworth, Liina Kiho, Marian Malone, Neil J Sebire

**Affiliations:** 1 Department of Histopathology, Camelia Botnar Laboratories, Great Ormond Street Hospital, Great Ormond Street, London WC1N 3JH, UK; 2 Institute of Child Health, UCL, London, UK

**Keywords:** Infant, Autopsy, Postmortem, Organ weights

## Abstract

**Background:**

Infancy is the most common period for childhood death, including both neonatal deaths from obstetric or medical complications and sudden unexpected infant deaths. The weighing of organs at autopsy is an established process and is recommended in current protocols. However, minimal contemporary data is available regarding reference ranges for organ weights of infants.

**Methods:**

Organ weight data for consecutive infant autopsies over a 14 year period performed at a single tertiary centre, including >1,000 cases, were examined in order to provide up to date reference ranges across this age range, using linear regression modelling and the standard LMS method.

**Results:**

1,525 infant autopsies were analysed, of which 1,190 were subsequently used in the creation of linear regression models prior to performance of the LMS method. Organ weight charts were produced for the 5th, 25th, 50th, 75th and 95th centiles for the heart, lungs, liver, spleen, kidneys, pancreas, thymus gland and adrenal glands.

**Conclusion:**

This study provides the largest single centre contemporary dataset of infant autopsies allowing provision of up-to-date ‘normal’ ranges for all major organ weights across this age range.

## Background

Autopsy currently remains the gold standard for determining the pathological process underlying the cause of death, based on anatomical dissection, macroscopic assessment of internal organs and sampling for histological examination and other ancillary investigations. Standard autopsy procedure includes weighing of internal organs and is recommended in current guidelines for investigation of Sudden Unexpected Death in Infancy [1].

The provision of normative data for the interpretation of such organ weights is available, but is often based on decades-old data or data pooled from several centres with possible variability in measurement and prosection [2,3]. For example, one study includes data from autopsies undertaken over a 15 year period across 35 counties in the United States [4]. More recent data has focused upon either specific organs, the apparent significance of ratios or values such as lung weight/body weight [5], or organ weights in selected clinical groups, some including sudden infant death syndrome [6]. Most studies have included less than 500 cases, though one publication reported a larger sample size of 553 autopsies, including fetuses, stillborn and liveborn infants [7].

The aim of this study is to provide contemporary reference ranges for visceral organs from a large series of consecutive infant autopsies, all performed at a single centre by specialist paediatric pathologists following a common protocol.

## Methods

All post-mortems performed at a single tertiary level, specialist paediatric centre using a standardised protocol over a 14 year period from 1996 to 2009 were reviewed and data entered into a specifically designed anonymised database. This included demographic details, autopsy findings, and organ weights recorded on digital or analogue calibrated scales, as well as macroscopic and microscopic appearances and results of ancillary investigations. Recording of the visceral organ weights includes the heart, lungs, thymus, liver, spleen, adrenals, pancreas and kidneys in all cases and selected additional organs as required.

Infants (children aged 365 days or younger) were identified and for each case, the macroscopic findings during the autopsy examination and subsequent histological findings were reviewed. Only unfixed weights were included, and for the purposes of this analysis organ weights were also excluded if there were macroscopic or histological findings affecting the organ which directly or potentially contributed to the death. This included findings such as tumour, haematoma, laceration, or necrosis (Table 1). Cases were evaluated based upon presentation and age.

**Table 1:** Examples of exclusion criteria that either lead to, or potentially could have caused death, based upon macroscopic and histological findings in 1,190 infant autopsy cases based upon differing organs

**Organ**	**Exclusion criteria on macroscopic findings**	**Exclusion criteria on histological findings**	**Cases included**
**Heart**	Myocardial Infarction, Ruptured ventricular aneurysm, Transplanted Heart, Cardiomyopathy	Myocarditis; rejection; extensive myocardial infarction	868
**Lungs**	Empyema, Laryngeal Stenosis/Atresia, Abscess, pulmonary hypoplasia as provided in the post-mortem report	TB, PCP Pneumonitis, Chronic Lung Disease, Asthma	764
**Liver**	Moderate to severe necrosis/infarction, tumour, haemorrhage	Acute or chronic hepatitis with necrosis, liver abscesses, neoplasia	1051
**Spleen**	Splenic laceration, haematoma	Abscess, malignancy or acute splenic sequestration	1101
**Kidneys**	Neoplasia, cystic dysplasia.	Neoplasia, dysplasia, cystic disease, glomerulonephritis or severe acute rejection.	1040
**Pancreas**	Neoplasia, haemorrhage, necrosis	Neoplasia, necrosis	1025
**Thymus**	Abscess or thymus encased in pus	Lymphoma, or Langerhans Cell Histiocytosis	1023
**Adrenals**	Abscesses, hypoplasia, necrosis or haemorrhage	Neoplasia, haemorrhage with necrosis	1039

Therefore, data were used for analysis for normal range calculation only from cases in which the pathologist undertaking the autopsy reported the organs as macroscopically normal and in which there were no significant histological abnormalities that contributed to the death. These exclusion criteria were used to provide organ weights for cases where significant pathological processes were not present and as such, were considered the best available ‘reference range’. Infants who were born prematurely (below 36 weeks of gestation) were excluded.

Statistical analysis for organ weights was first performed using IBM SPSS Statistics Version 21.0 (Released 2012, IBM Corp. Armonk, New York, USA) to evaluate significant differences for gender and age by using stepwise linear regression modelling. If gender was statistically significant for the organ weight (p <0.05), cases were separated. Cases then underwent linear regression analysis for age, with the creation of z-scores (defined as the number of standard deviations an observation is above or below the mean). Prior to constructing the centiles, cases which were two or more standard deviations from the mean were excluded, to avoid the influence of outliers, which may have resulted from erroneous recording at the time of autopsy. The application of this approach to all data provided a standardised technique to avoid observer bias. Furthermore, post-mortem interval was assessed for all organs.

Subsequently, analysis of the remaining organ weight values were performed using LMS Chartmaker Light (Version 2.54, Medical Research Council, UK), with provision of z-scores and centile charts using the standard LMS Method (Table 2) [8]. This method is previously well-described and has been used to calculate the World Health Organization (WHO) growth charts [9]. It allows fitting of smooth centile curves to reference data, by normalising data through power Box-Cox transformation of the skew (L), the median (M), and the generalized coefficient of variation (S). This allows the skewness, median and variability of the reference data to vary with age and has been used in creating fetal [10] and childhood growth centile charts to allow continuous changes of weight and other variables [9,11].

**Table 2:** Best fit statistical models for formulation of centile charts, plotted by organ with deviance and values for L (skewness), M (median) and S (coefficient of variation) using the LMS method

**Organ**	**Gender**	**Penalized deviance**	**L**	**M**	**S**
**Heart**	Male	3051.9	3	4	3
	Female	1935.1	3	4	3
**Combined lungs**	Male	3781.1	3	4	3
	Female	2706.1	3	4	3
**Liver**	Male	5866.4	3	4	4
	Female	4253.9	3	4	4
**Spleen**	Male	3894.1	3	4	3
	Female	2863.7	2	3	2
**Combined kidneys**	Male	4296.2	3	4	4
	Female	2959.4	3	4	4
**Pancreas**	Male	2595.6	3	4	4
	Female	1846.4	3	4	4
**Thymus**	Male	4219.5	3	4	3
	Female	2862.4	3	4	3
**Combined adrenals**	Male and Female	3635.2	3	4	4

The study was approved by the local LREC (London (Bloomsbury) National Research Ethics Service Committee; formerly Great Ormond Street and Institute of Child Health Research Ethics Committee), as part of a larger review of paediatric autopsy findings.

## Results

Following analysis of the overall 1,525 autopsy reports, 1,190 (77.9%) cases were included for analysis. This included 605 (39.6%) term infants and 585 (38.4%) cases with no provided gestational age. 335 documented (21.9%) premature infants were excluded. There was no statistically significant difference between the cohorts who were known to be born at term and those in whom no gestational age was specifically provided, suggesting that the majority of preterm cases were documented as such and excluded. Of the remaining 1,190 cases, each organ was individually evaluated and excluded if there were findings that would be either a definite or potential cause of death (Table 1). Post-mortem interval only had statistically significant effect on combined lung weight (p = 0.034), however further independent analysis of the right and left lung weights confirmed post-mortem interval only had a significant influence on the right lung weight (p = 0.023) and therefore was not included as a variable for the overall combined lung weight. Of the 1,190 cases, only 43 (3.6%) had parental ethnic origins accurately provided and of these, only 30 (2.5% of the total) had both parents ethnic origin; hence ethnicity could not be accounted for in the models.

Analysis of remaining organ weights (as stated in Table 1) was undertaken based upon only valid data and utilised LMS scores with provision of penalized deviance (Table 2) using the selected cases (Table 3). Production of centile prediction charts was performed showing the 5th, 25th, 50th, 75th and 95th centiles for the heart, liver, thymus, spleen, pancreas and combined kidneys and lungs for boys and girls respectively (Figures 1, 2, 3, 4, 5, 6, 7, 8, 9, 10, 11, 12, 13 and 14) as well as a combined adrenals weight chart for boys and girls (Figure 15).

**Table 3:** Numbers of cases selected for analysis by gender, including initial cases and subsequent cases used for centile analysis, within 2 standard deviations from the mean, following linear regression modelling

**Organ**	**Gender**	**Total cases**	**Cases within 2 standard deviations**	**Total cases analysed for centiles**
**Adrenal**	Both	1039	998	998
**Heart**	Female	356	337	824
	Male	512	487	
**Combined lungs**	Female	320	306	726
	Male	444	420	
**Liver**	Female	447	423	997
	Male	604	574	
**Kidneys**	Female	444	421	999
	Male	596	578	
**Spleen**	Female	468	461	1067
	Male	633	606	
**Pancreas**	Female	436	414	973
	Male	589	559	
**Thymus**	Female	428	407	970
	Male	595	563	

**Figure 1:**
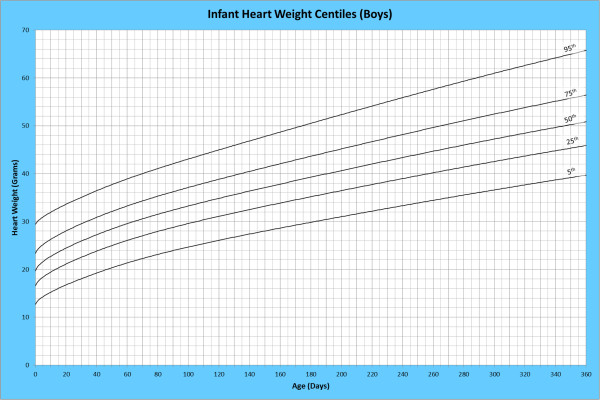
Infant heart weight centiles (boys).

**Figure 2:**
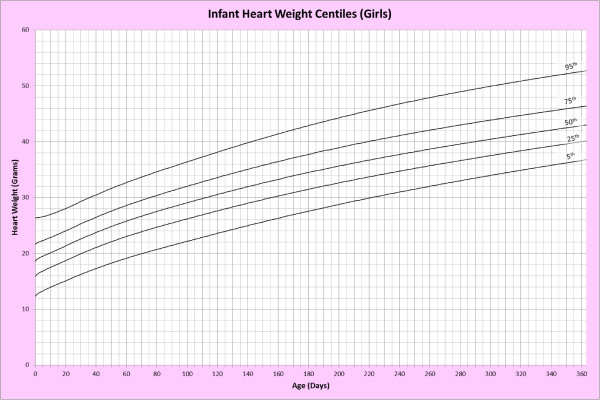
Infant heart weight centiles (girls).

**Figure 3:**
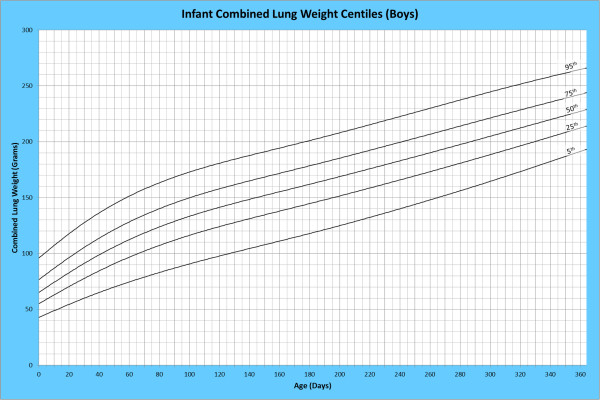
Infant combined lung weight centiles (boys).

**Figure 4:**
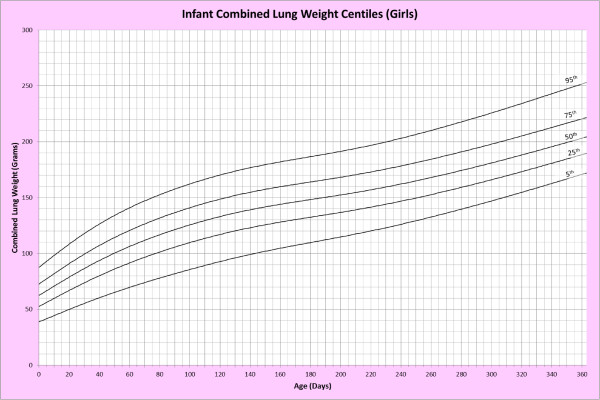
Infant combined lung weight centiles (girls).

**Figure 5:**
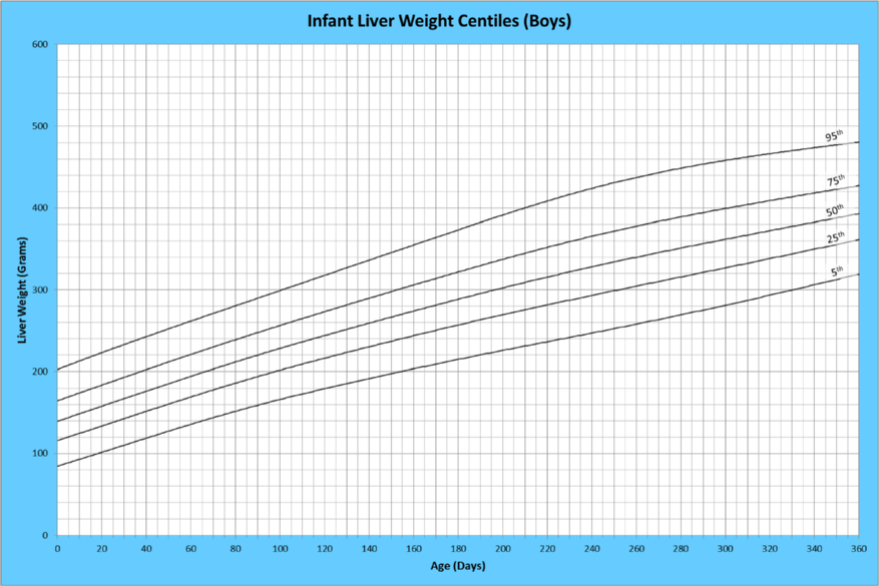
Infant liver weight centiles (boys).

**Figure 6:**
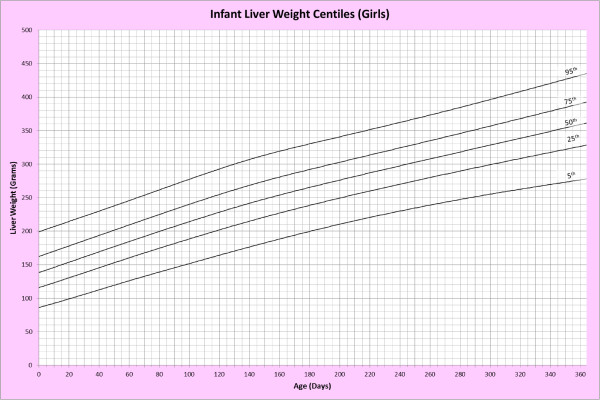
Infant liver weight centiles (girls).

**Figure 7:**
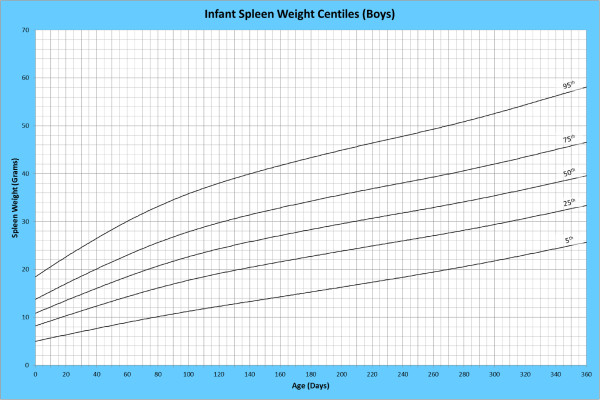
Infant spleen weight centiles (boys).

**Figure 8:**
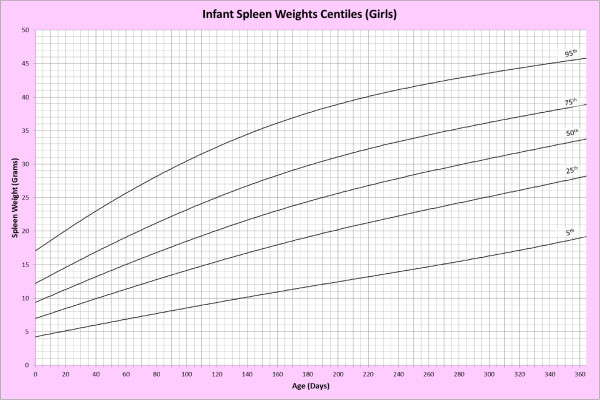
Infant spleen weight centiles (girls).

**Figure 9:**
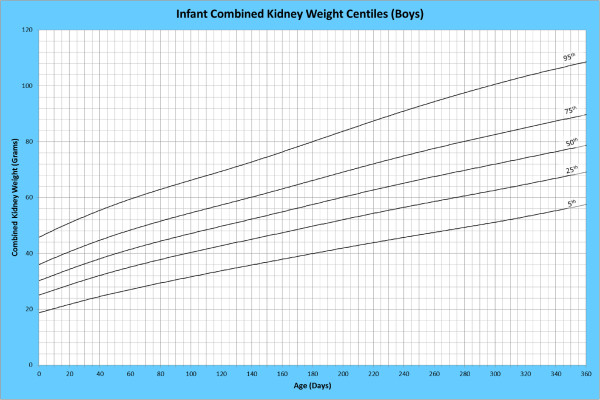
Infant combined kidney weight centiles (boys).

**Figure 10:**
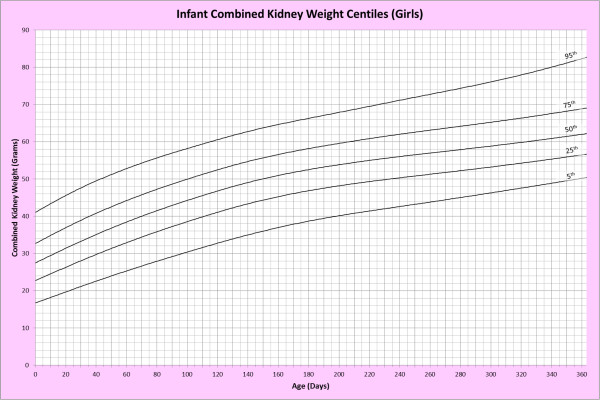
Infant combined kidney weight centiles (girls).

**Figure 11:**
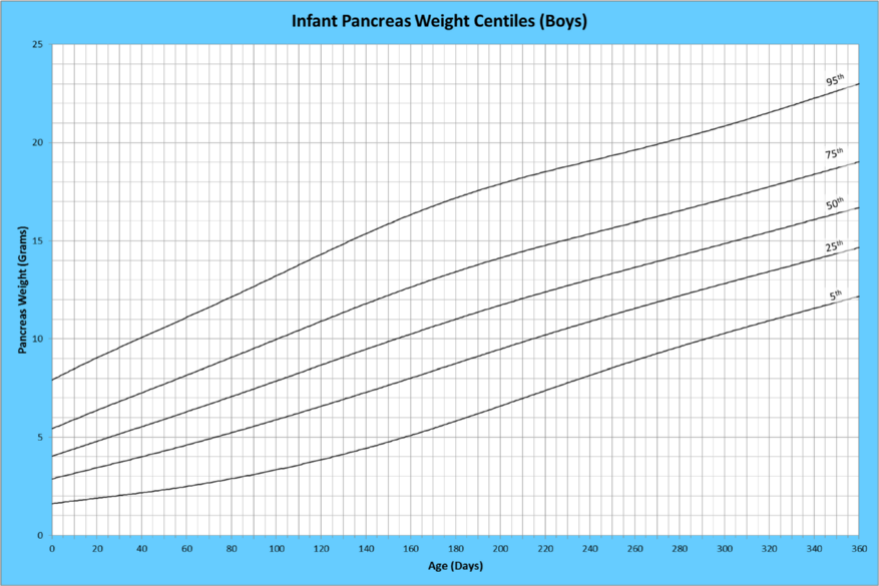
Infant pancreas weight centiles (boys).

**Figure 12:**
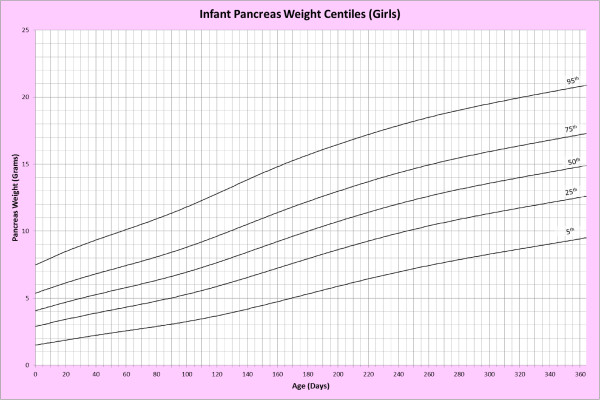
Infant pancreas weight centiles (girls).

**Figure 13:**
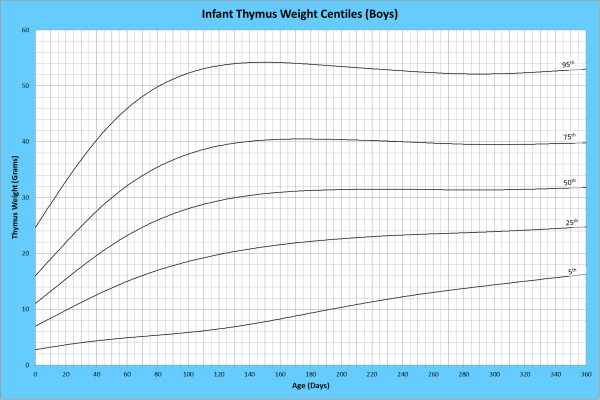
Infant thymus weight centiles (boys).

**Figure 14:**
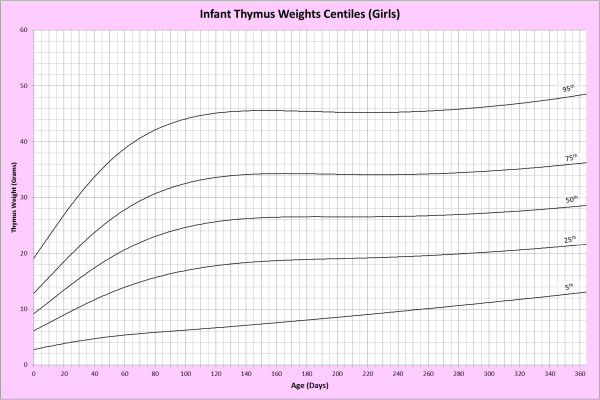
Infant thymus weight centiles (girls).

**Figure 15:**
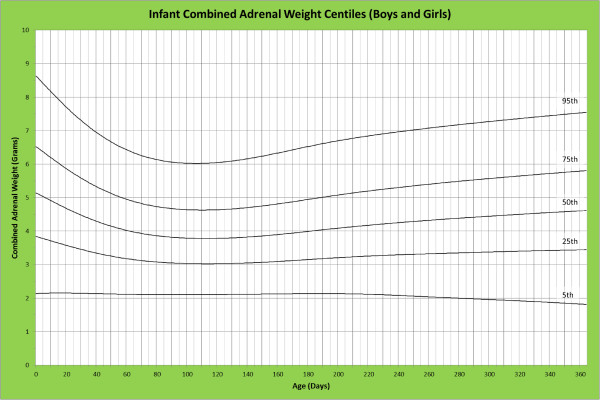
Infant combined adrenal weight centiles (boys and girls).

## Discussion

The findings of this study provide contemporary autopsy reference ranges for infant organs, by age, during the first year of life. This data is based on a large number of cases (over 1,000 infant autopsies), all derived from a single centre using the same methods and autopsy techniques. All autopsies were carried out according to a standard protocol and organs weighed using the same calibrated, paediatric-specific equipment, and as far as is possible in this clinical setting, only macroscopically and microscopically normal organs were included to determine ranges. This is the largest series providing such data on infant weights and is the only series to look at and produce centile charts based on gender where significant.

The strict exclusion criteria used for each organ and the large number of cases included, allow provision of the current best possible estimates of ranges for ‘normal’ organ weights across this important age range. It remains part of the standard autopsy protocol that weights are obtained for all major organs, although the clinical significance of organ weights for determining cause of death in this infant population remains uncertain. Previously published studies have examined the relationship of organ weights with specific pathological processes [12,13] but with variable findings [6]. This dataset will allow subsequent comparative studies to be performed and should provide a valuable reference for practising pathologists requiring reference ranges for organ weights derived contemporaneously.

It should be clearly understood that these charts are not normal ranges designed for the assessment of live infants, but rather to provide a range of centiles for which specific organs can be compared to previous cases without significant pathology in the context of infant autopsy. In this context, organ weights below the 5th or above the 95th centiles may indicate to the pathologist that there may be a significant pathological process.

Great Ormond Street Hospital for Children is a specialist referral centre for the investigation of a wide variety of paediatric disorders. The department of Pathology undertakes autopsy examination of paediatric deaths on behalf of many Coronial offices from London and the surrounding areas, as well as undertaking both forensic paediatric autopsy examinations and consented non-Coronial autopsies. The majority of the caseload included in this series therefore represents cases examined on behalf of Her Majesty’s Coroner, most of which presented as sudden unexpected death in infancy (SUDI). In view of the nature of such referrals, many clinical details such as precise gestational age at birth and specific ethnic background of the parents are not provided in a substantial proportion of cases and hence could not be formally accounted for in our models; for example no information regarding exact gestational age at birth, was available in 38% of cases. Nevertheless, it should be noted that this information is also unavailable for the existing previous datasets used by most institutions worldwide for normal ranges for organ weights and is significantly offset in the present study by the much larger number of cases included. Furthermore, in this study we excluded those of known preterm delivery, and of the remaining cases there was no significant difference in distribution of organ weights between those with or without known gestations, validating their inclusion. In addition, the main geographic area served, London and surrounding areas, represents a highly diverse and multicultural population and hence the data are derived from infants from a range of ethnic groups. Such methodological issues simply cannot be controlled for in post-mortem population based studies unless huge numbers of cases are included, which then introduces other significant areas of variation from which these data do not suffer. For example, the use of pooled data from numerous centres is associated with differences in method of dissection prior to weighing through to systematic bias in weighing instruments used. The main aim of this data is to provide contemporary expected normal post-mortem organ weights for age in infancy and to provide ranges which can be used to identify clearly pathological processes; any variance in organ weights due to the factors noted above are therefore likely to be of little significance in routine clinical practice. Finally, it should again be noted that similar methodological issues affect existing historical datasets, many of which are based on data derived many decades previously. It is well reported that infant weights [14,15] have increased significantly and provision of such contemporaneous data allows more accurate and relevant normal ranges for use in current clinical practice.

There are intrinsic issues with deriving normal ranges for organ weights from infant autopsies, affecting this and all previous datasets, not least of which is the fact that a true ‘normal’ control group is unavailable, since entirely ‘normal’ infants do not undergo autopsy examination. Even if no definite cause of death is identified, such as in unexplained SUDI, this does not exclude underlying pathophysiological processes which may have affected organ weights. Non-specific autopsy findings related in part to changes after death, such as congestion and oedema, could affect organ weight but may not provide diagnostic clues towards the mode or cause of death. Nevertheless, by excluding cases of congenital abnormality or significant macroscopic or microscopic pathologies affecting such organs in the present study, it is possible to provide the best estimates of normal organ weight ranges in this setting.

The rationale for recording organ weights at autopsy is that deviations from normal may provide additional evidence in supporting an abnormal morphological finding, particularly in the prosection room itself, allowing the selection of appropriate samples for further investigation. The utility of organ weight as a marker of disease in this context is relatively poorly evidence-based, although for some conditions, such as myocarditis and cardiomyopathy, age-related heart weights may provide useful clues to the underlying diagnosis, with variable accuracy depending on the precise disease type and severity [16,17]. By the use of contemporary weight ranges, future studies may provide additional information regarding the specific utility of organ weight deviations in a range of paediatric conditions, which may also add to our knowledge of the underlying pathophysiological mechanisms present.

## Conclusion

In summary, this study provides the largest single centre contemporary dataset of infant autopsies all performed to a standard protocol by paediatric pathologists, allowing provision of up-to-date ‘normal’ ranges for all major organ weights across this age range. Such data may be of use for both generating autopsy reports in clinical practice and also for future studies examining the role of organ weights in disease.

## Abbreviation

SUDI: Sudden unexpected death in infancy.

## Competing interests

The authors declare that they have no competing interests.

## Authors’ contributions

JWP and NJS conceived of, and coordinated the study. JWP, ARB, NJS participated in its design. JWP, MTA, MM, LK and NJS participated in acquisition/interpretation of data. JWP performed the statistical analysis. All authors helped to draft the manuscript. All authors read and approved the final manuscript.

## Pre-publication history

The pre-publication history for this paper can be accessed here:

http://www.biomedcentral.com/1472-6890/14/18/prepub
